# MACNet: A Multidimensional Attention-Based Convolutional Neural Network for Lower-Limb Motor Imagery Classification

**DOI:** 10.3390/s24237611

**Published:** 2024-11-28

**Authors:** Ling-Long Li, Guang-Zhong Cao, Yue-Peng Zhang, Wan-Chen Li, Fang Cui

**Affiliations:** 1 Guangdong Key Laboratory of Electromagnetic Control and Intelligent Robots, College of Mechatronics and Control Engineering, Shenzhen University, Shenzhen 518060, China; 2150296002@email.szu.edu.cn; 2 Shenzhen Institute of Information Technology, Shenzhen 518172, China; ypzhang@sziit.edu.cn; 3 School of Psychology, Shenzhen University, Shenzhen 518060, China; lli1221@126.com

**Keywords:** electroencephalogram (EEG), motor imagery classification, CNN, attention mechanism

## Abstract

Decoding lower-limb motor imagery (MI) is highly important in brain–computer interfaces (BCIs) and rehabilitation engineering. However, it is challenging to classify lower-limb MI from electroencephalogram (EEG) signals, because lower-limb motions (LLMs) including MI are excessively close to physiological representations in the human brain and generate low-quality EEG signals. To address this challenge, this paper proposes a multidimensional attention-based convolutional neural network (CNN), termed MACNet, which is specifically designed for lower-limb MI classification. MACNet integrates a temporal refining module and an attention-enhanced convolutional module by leveraging the local and global feature representation abilities of CNNs and attention mechanisms. The temporal refining module adaptively investigates critical information from each electrode channel to refine EEG signals along the temporal dimension. The attention-enhanced convolutional module extracts temporal and spatial features while refining the feature maps across the channel and spatial dimensions. Owing to the scarcity of public datasets available for lower-limb MI, a specified lower-limb MI dataset involving four routine LLMs is built, consisting of 10 subjects over 20 sessions. Comparison experiments and ablation studies are conducted on this dataset and a public BCI Competition IV 2a EEG dataset. The experimental results show that MACNet achieves state-of-the-art performance and outperforms alternative models for the subject-specific mode. Visualization analysis reveals the excellent feature learning capabilities of MACNet and the potential relationship between lower-limb MI and brain activity. The effectiveness and generalizability of MACNet are verified.

## 1. Introduction

Brain–computer interface (BCI) is a cutting-edge technology that enables the direct communication between the human body and external intelligent devices. This can be achieved by decoding brain activities to recognize human intent, with broad applications ranging from healthcare to assisted living [1,2,3]. Electroencephalograms (EEGs) are among the most popular inputs for BCI systems given their advantages of low cost, portability, high temporal resolution, and a lack of side effects [4]. Motor imagery (MI) belongs to spontaneous paradigm for EEG-based BCI systems that generates endogenous EEG signals by imagining the intended motion without an external stimulus. This paradigm is more natural and intuitive for controlling smart equipment than other evoked paradigms such as steady-state visual evoked potential (SSVEP) and the P300, making MI-based BCI more user-friendly for individuals both with and without disabilities. MI-EEG signals are widely utilized in medical and nonmedical industry applications, such as stroke rehabilitation, gaming, virtual reality (VR), and robot control [4,5,6]. MI-EEG-based BCIs have garnered significant attention for their potential to bridge brain activity and machine actions in a natural and noninvasive manner [7,8]. In the medical field, MI-EEG supports neurorehabilitation, enabling motor recovery and robotic prosthesis control for stroke patients. In nonmedical fields, MI-EEG enhances VR immersion and improves human–computer interaction, making it pivotal for BCI applications in diverse industries. However, the growth of BCIs and rehabilitation engineering is restricted by the limited decoding performance and generalizability of MI-EEG signals [6]. One major challenge for MI-based BCI applications is accurately recognizing human motion intention (HMI) from EEG signals because MI-EEG signals have a low signal-to-noise ratio (SNR), nonlinearity, high complexity, and high variability [7,8]. Currently, traditional machine learning (ML) and deep learning (DL) methods are widely used for decoding the EEG signals of upper-limb and lower-limb MI. 

ML-based approaches consist of the feature extraction, feature selection, and classification stages [7,9]. Common feature extraction methods use features from the time, frequency, time–frequency, and spatial domains [7]. The most commonly used feature extraction methods include power spectral density (PSD) features and the common spatial patterns (CSP) algorithm [10,11]. Linear discriminate analysis (LDA) [12,13] and support vector machines (SVMs) [14,15] are widely applied in the MI classification stage [7]. Among them, the filter bank common spatial pattern (FBCSP) and its variants achieved superior performance in MI classification [6]. Note that conventional ML-based methods rely on manual feature extraction, which requires specific prior knowledge. The separate structure of feature extraction and classifier may cause imperfect optimization [10,16,17].

Conversely, DL-based methods with end-to-end frameworks facilitate the development of MI-EEG decoding due to their exceptional representation capability [8]. Convolutional neural networks (CNNs) specialize in extracting local features and possess powerful pattern classification abilities. Compared with other DL architectures (e.g., autoencoders, deep belief networks, and recurrent neural networks) [4], CNNs and hybrid-CNN variants are the most widely used and have demonstrated superior performance in MI-EEG classification. However, CNNs with local receptive fields face challenges in extracting deeper and global dependencies [17], which impacts decoding performance and generality. The attention mechanism, on the other hand, shows promise in capturing the long-term correlations of EEG features and selectively focuses on the most salient parts. Attention mechanisms such as squeeze-and-excitation (SE) [18], convolutional block attention module (CBAM) [19], and multihead attention-based transformers [20] have made significant progress in the fields of computer vision and natural language processing. 

Recent studies have introduced attention mechanism to CNNs to improve MI classification [3,6,8,11,17,21,22,23,24,25,26]. For example, Altuwaijri et al. [22] utilized multibranch SE attention modules as intermediate layers in three CNN-based blocks for MI-EEG classification. The SE modules acted as gating mechanisms across the channel dimension and their efficacy was validated using the BCIC-IV 2a and HGD datasets. Yu et al. [26] incorporated parallel CBAM into a feature fusion network, enhancing feature fusion quality and making the feature maps more discriminative. The classification performance was subsequently improved through validation on the BCIC IV 2a dataset. Song et al. [17] combined a CNN with a transformer (Conformer) to capture local and global EEG features; the self-attention module extracted global dependencies from temporal features. Conformer demonstrated excellent classification performance on three public datasets (MI and emotion recognition). However, the model’s complexity and efficiency require further improvement for MI-BCI applications. Overall, the attention mechanism shows promise for improving MI-EEG decoding performance.

Research on MI classification represents recognizing HMIs, and it is primarily focused on upper-limb MI [23]. However, research on lower-limb MI decoding is as significant as that on upper-limb MI decoding in BCI [27], as it plays a pivotal role in applications, such as the gait rehabilitation for individuals with motor impairments, prosthetic control for amputees, and mobility restoration [28]. Peng et al. [27] emphasized the critical importance of lower-limb MI decoding for these applications. Despite its importance, relatively less research has been conducted on EEG decoding for HMIs of the lower limb than for those of the upper limb [29]. 

**Figure 1:**
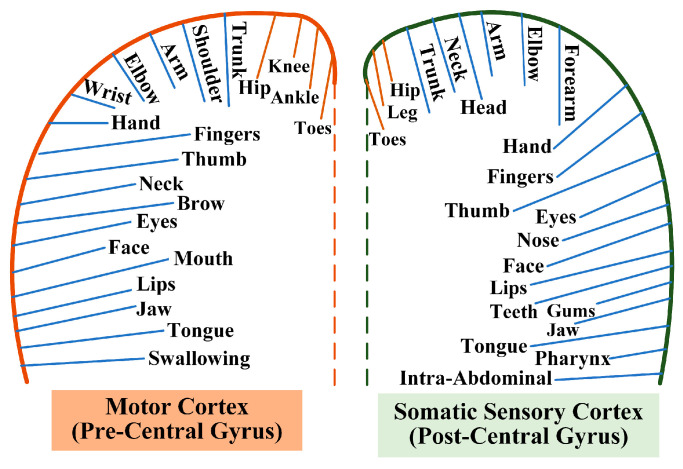
Map of functional areas of brain regions for motor tasks.

Research on lower-limb MI has two limitations. First, the cortical representation of lower-limb motions (LLMs), including MI, is more localized and concentrated in the medial aspects of the motor and sensory cortex than the behaviors of the upper limb [30]. This anatomical difference results in a narrower and smaller cortical activation area for the LLMs, as illustrated in Figure 1. Lower-limb MI signals exhibit distinct characteristics, including lower signal intensity and a more restricted spatial distribution, resulting in reduced spatial resolution. Additionally, the activation associated with lower-limb MI tasks tends to be deeper in the brain and less dispersed, contributing to more complex and weaker signal expression [31]. The limited cortical activation for lower-limb MI, coupled with the weaker signal intensity, poses challenges in accurately capturing the critical neural information necessary for decoding lower-limb MI tasks. The weaker signals are more susceptible to noise, and the low SNR makes it more difficult to differentiate meaningful brain activity from background noise, further complicating lower-limb MI classification [32]. This issue is exacerbated by the fact that lower-limb MI signals often exhibit more subtle temporal dynamics and lower spatial resolution than upper-limb MI signals do. These unique spatial and temporal characteristics of lower-limb MI signals necessitate the use of advanced techniques, such as attention mechanisms for spatiotemporal feature extraction, to address the complex spatial and temporal dynamics effectively and improve classification performance. Second, there is a lack of public datasets for lower-limb MI. MI-EEG signals are typically obtained from diverse public EEG datasets, which predominantly focus on common MI tasks from the upper limbs [4]. However, decoding MI-EEG signals from lower limbs faces the challenge of scarce available data, and less data may impact the ability of DL-based models to effectively learn discriminative patterns. Consequently, this paucity of data makes identifying lower-limb neural activity more challenging than identifying upper-limb neural activity, which requires further research [27,33]. The lower-limb MI-EEG signals exhibit low SNR and complex spatiotemporal correlations, and the abovementioned CNN-based methods with attention mechanisms primarily consider the temporal dimension for MI classification. However, these approaches often overlook the global dependencies across temporal, spatial, and channel dimensions, which are critical for capturing the full range of EEG features. This limitation may hinder the ability of the EEG decoding model to extract relevant information fully, leading to suboptimal classification performance. In contrast, multidimensional attention mechanisms can enhance a model’s ability to recognize complex activities and extract essential features from sensor data [34].

To address these challenges, this paper proposes a multidimensional attention-based CNN, termed MACNet, which integrates attention mechanisms across temporal, spatial, and channel dimensions to improve the decoding performance of lower-limb MI-EEG signals. The model integrates a temporal refining module and an attention-enhanced convolutional module to improve feature extraction capabilities. Specifically, MACNet is designed for lower-limb MI decoding, utilizing an EEG dataset tailored for lower-limb MI tasks. MACNet exploits the merits of both the CNN’s hierarchical feature extraction and attention mechanisms, leading to superior classification performance in lower-limb MI-EEG decoding compared with state-of-the-art (SOTA) models. The temporal refining module addresses the inherent low SNR of EEG signals by employing self-attention mechanism to concentrate on the most distinctive temporal information while suppressing noise. By refining the raw EEG signals, the temporal refining module enhances the signal quality before further processing. The refined EEG signals are subsequently fed into the attention-enhanced convolutional module to capture the spatiotemporal characteristics of the EEG data, which learns ‘what’ the most meaningful features are and ‘where they are located from the temporal, channel, and spatial dimensions, to obtain highly recognizable features. Finally, a classifier with a convolutional layer and SoftMax function outputs the decoding results. The main contributions of this study are threefold and can be summarized as follows:

(a) A MACNet model is proposed to decode lower-limb MI signals, which leverages the local and global feature representation abilities of CNNs and attention mechanisms and significantly improves the classification performance for multiple lower-limb MI tasks.
(b) To address the low SNR of the lower-limb MI-EEG signals, the temporal refining module adaptively investigates the critical temporal information of each electrode channel to refine the EEG signals. Additionally, the model incorporates a lightweight CBAM that further enhances feature extraction by refining feature maps along both the channel and spatial dimensions, ensuring the full exploitation of the spatiotemporal characteristics of the EEG signals.
(c) A specified lower-limb MI experiment with four routine LLMs is designed to enrich and deepen the research on decoding lower-limb MI. A lower-limb MI dataset with 10 subjects over 20 sessions is built, providing sufficient samples for training and testing, offering a solid foundation for future investigations in this domain.
(d) To verify the effectiveness of MACNet, extensive experiments are conducted for comparison, and the effects of each module are investigated. MACNet achieves SOTA classification performance on the lower-limb MI dataset and a public EEG dataset, validating its efficacy in addressing the challenges of lower-limb MI-EEG decoding.

## 2. Related Work

### 2.1. DL-Based MI-EEG Decoding 

The success of DL has greatly advanced the development of EEG decoding in MI-based BCIs [10,17]. Inspired by the FBCSP, Schirrmeister et al. [35] first proposed the following two well-known EEG decoding models: shallow ConvNet, which is specifically designed for MI-EEG signal classification; and deep ConvNet, which has a general-purpose architecture suitable for different feature types. Both models achieve competitive classification performance compared with the FBCSP algorithm on various datasets. Mane et al. [36] proposed an advanced MI decoding algorithm named the filter-bank convolutional network (FBCNet), which combines the neurophysiological priors of the FBCSP with CNNs. FBCNet provides multiview EEG representations of spatial features of different frequency bands and temporal features, resulting in remarkable classification performance across four public MI datasets. Lawhern et al. [37] developed a compact CNN (EEGNet) for EEG-based BCIs, which incorporated depthwise and separable convolutions into the network. EEGNet can learn frequency and spatial features intra- and interchannels, being a general EEG decoding model for different BCI paradigms, such as MI, the P300, error-related negativity responses, and movement-related cortical potentials (MRCPs). Ingolfsson et al. [38] proposed an EEG-TCNet model for MI classification. The model combined EEGNet with a temporal convolutional network (TCN) to supplement and utilize EEG features from the temporal domain. TCNet achieved outstanding accuracy while requiring few training parameters. Based on the TCNet, Altaheri et al. [6] designed an attention-based TCN (ATCNet), with multihead self-attention (MSA) embedded as intermediate layers in the TCNet. ATCNet obtained average classification accuracies (ACAs) of 85.38% and 70.97% for subject-dependent and subject-independent experiments, respectively, on the BCIC IV 2a dataset, representing one of the highest performances to date from comprehensive aspects. Liu et al. [5] proposed a CNN-based network with temporal and channel attention mechanisms for MI-EEG classification, demonstrating that the salience level features of EEG signals from time slices and electrode channels could be captured for further classification. Luo et al. [23] proposed a shallow mirror transformer (SMT) for MI-EEG classification; the framework was similar to Conformer [17] but had only one MSA layer. SMT obtained ACAs of 70.41% and 67.28% for the within-subject and cross-subject experiments, respectively, on the BCIC IV 2a dataset.

In general, incorporating an attention mechanism into a CNN has proven to be effective for MI-EEG classification by addressing ‘what’, ‘where, ‘when’, and ‘which’ aspects require attention, namely, the channel mechanism, spatial mechanism, temporal mechanism, and branch mechanism, respectively. Although current studies have yielded notable results in classifying MI-EEG signals, there is still room for improving the decoding performance of MI-based BCIs [6].

### 2.2. Decoding of Lower-Limb Motion Intention 

Recognizing lower-limb motion intention (LLMI) is highly important in BCI-based medical and nonmedical applications, such as stroke rehabilitation and motion auxiliary applications [1]. MI directly reflects LLMIs with spontaneous EEG generation. Some studies have focused on decoding upper-limb MI to map predefined LLMIs for BCI applications, such as using the decoding results of MI of left- or right-hand movement to indirectly represent LLMIs [39,40,41,42]. However, decoding lower-limb MI directly is more acceptable and user-friendly during LLMI recognition. Several attempts have been made to explicitly decode lower-limb MI. The commonly used lower-limb MI tasks are left- and right-leg movements, foot movements, stepping, walking, standing, sitting, and standing up [27,30,33,40,43,44,45,46,47,48,49]. Bulea et al. [43] proposed a Gaussian mixture model to classify three MI tasks—stand-up, sit-down, and quiet states—from EEG signals, achieving an ACA greater than 70.00%. Jeong et al. [46] proposed a subject-dependent and section-wise spectral filtering method to extract MRCP features, with a binary regularized LDA to classify walking intention and rest state, which obtained an ACA of 86.00%. Peng et al. [27] utilized a particle swarm optimization-based SVM to classify the MI of the leg lifting and rest state based on multidomain fusion features. Ma et al. [30] designed a compound-limbs EEG paradigm that integrated upper-limb swing with lower-limb stepping to decode stepping forward intention. CSP-based methods were adopted for feature extraction and three ML-based methods (SVM, K-nearest neighbor, and RF) were used for classification. The best ACA reached 73.70 ± 12.47% under MI conditions. Wei et al. [33] proposed three sub-band cascaded CSP algorithms to extract common EEG features across different subjects, and deep transfer learning-based AlexNet was used as the classifier, which realized the cross-subject MI classification of the following three MI tasks: left stepping, right stepping, and idle state. Zhang et al. [49] combined traditional ML with DL techniques to form a shallow and deep ensemble method for classifying lower-limb MI across the left leg, right leg, and rest. The shallow part included wavelet transformation and FBCSP, while the deep part included CNN, MSA, and TCN. The proposed method achieved an ACA of 60.27% for three classes and 64.20% for two classes (lower-limb MI vs. rest).

In summary, ML-based techniques, such as the CSP approach, which requires manual feature extraction, are predominantly utilized for lower-limb MI classification. In contrast, DL-based methods for lower-limb MI decoding are gradually being explored and are gaining increasing attention.

### 2.3. Multidimensional Attention-Based Network

The concept of multidimensional attention has garnered significant interest across various domains, including EEG signal processing, machine life prediction, speech separation, human activity recognition, and status recognition. It allows models to capture and focus on relevant features from different perspectives, such as temporal, spatial, and channel dimensions. Several studies have explored the application of multidimensional attention mechanisms, leading to substantial improvements in performance across a range of tasks. Xu et al. [50] proposed an attention-based multiple dimensional EEG transformer for emotion recognition, which extracted 3D spectral-spatial-temporal features from EEG signals and achieved a competitive recognition performance. Similarly, Xie et al. [51] designed a CNN with a 3D attention mechanism to obtain synthetic degradation features in machine remaining useful life prediction. In the domain of speech separation, Li et al. [52] proposed a multidimensional attention fusion network, which extracted information of narrow and full bands along temporal, spatial, and channel dimensions and maximized the utilization of different attention features. Liang et al. [34] proposed a 3D weight attention module that considers both spatial and weight information and could be integrated with convolutional models to enhance model performance without increasing computational burden for human activity recognition. Liu et al. [53] proposed an improved 3D CNN with a separable structure and multidimensional attention for welding status recognition, and the multidimensional attention mechanism compensated for accuracy. 

These studies demonstrate the broad applicability and effectiveness of multidimensional attention mechanisms across various fields. EEG signals have low SNR and exhibit high intersubject variability. This paper leverages multidimensional attention across temporal, spatial, and channel dimensions and proposes MACNet to address the inherent challenges of the noisy and complex lower-limb MI-EEG signals. Extracting meaningful features from EEG signals is crucial, as it improves classification accuracy and model generalization.

## 3. Proposed MACNet

**Figure 2:**
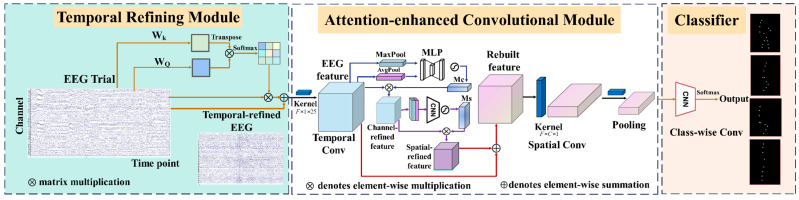
Overall architecture of the proposed model.

The proposed MACNet model includes a temporal refining module, an attention-enhanced convolutional module, and a convolutional classifier, as illustrated in Figure 2. The EEG signals are preprocessed and input into MACNet for MI classification.

MACNet is designed as an end-to-end model that automatically refines and extracts features from EEG signals through multidimensional attention mechanisms, removing the need for manual feature engineering. This proposed model differs significantly from existing EEG-based attention mechanisms by integrating attention across temporal, spatial, and channel dimensions. Existing models, such as CBAM, focus on features from spatial and channel dimensions, whereas models such as ATCNet and Conformer prioritize temporal dependencies. These approaches have a limited consideration of the full spatiotemporal correlations inherent in MI-EEG signals. Our MACNet model performs feature extraction and refinement across temporal, channel, and spatial dimensions. The input EEG signals are initially passed through the temporal refining module, which applies a self-attention mechanism to learn the temporal dependencies of each electrode channel. The attention map generated by the temporal refining module highlights salient temporal features while attenuating irrelevant noise, thus mitigating the low-SNR issue associated with EEG data. Next, the temporally refined EEG signals are processed using the attention-enhanced convolutional module, which sequentially applies temporal convolution, CBAM, and spatial convolution. The temporal convolution layer extracts high-level temporal features relevant to the specified frequency ranges relevant to MI. CBAM is then employed to adaptively refine features in the feature maps by selectively enhancing informative features along both the channel and spatial dimensions. Then, spatial convolution is performed to further extract spatial features between electrode channels. Specifically, MACNet integrates convolutional layers for local feature extraction, leveraging their ability to capture fine-grained temporal and spatial dependencies. CBAM strengthens global feature representation by adaptively weighing feature channels and refining spatial patterns. This synergy allows MACNet to capture the complex spatiotemporal dependencies inherent in EEG data effectively. Finally, an efficient classifier composed of a classifying convolutional layer and a SoftMax function is adopted to output the classification result. The detailed structure of MACNet, including modules, kernel size, output shape, and parameter setting, is provided in Table 1. 

### 3.1. Temporal Refining Module

Learning global temporal dependencies from EEG signals significantly improves the decoding accuracy in MI classification [17]. Within this context, a self-attention mechanism within the temporal refining module is employed to capture long-term dependencies of the EEG signals of each channel in the temporal dimension. This mechanism allows the model to discern the most relevant temporal features while minimizing the influence of irrelevant or noisy information. The temporal refining module effectively filters out noise and enhances the quality of the raw EEG signals, thereby addressing the challenge of low SNR and improving the data quality. As introduced in [20], for the self-attention mechanism, the self-attention layer consists of three components, query (***Q***), key (***K***), and value (***V***), where ***Q*** and ***K*** are obtained as follows:

(1) $$Q=W_{Q}X\in R^{C\times T}$$

(2) $$K=W_{K}X\in R^{C\times T}$$

where ***X*** is the input sequence. The attention map can be calculated as follows:

(3) $$\mathrm{Attention}(Q,K,V)=Soft\max(\frac{QK^{T}}{\sqrt{d}})V$$

where *d* represents the length of keys and queries, and ***V*** is defined as the input sequence. This design allows the raw input information to be preserved and dynamically refined by attention weights. In this context, a single-head self-attention with a head dimension of 61 is employed to calculate the attention score. Then, the temporally refined EEG signals are computed as follows:

(4) $$\overset{\wedge }{X}=A\mathrm{ttention}(Q,K,V)$$

To enhance the representation ability and avoid losing information, residual connection is used, and the output $\overset{\wedge }{X}$ is added to the input sequence to obtain the final temporally refined EEG trials $\tilde{X}$, which is given as follows:

(5) $$\tilde{X}=X+\overset{\wedge }{X}$$

### 3.2. Attention-Enhanced Convolutional Module

EEG signals represent neural activity and are characterized by both temporal coherence and interchannel relationships, making it critical to capture information across these dimensions in EEG decoding. Inspired by [19,35], we designed an attention-enhanced convolutional module that integrates a temporal convolutional layer, the CBAM, and a spatial convolutional layer to address the challenge of decoding complex spatiotemporal dependencies of EEG signals. 

The temporal convolution is operated over the time dimension via 40 filters with a kernel size of (1, 25) and a stride of (1, 1), allowing it to extract temporal features corresponding to frequencies above 10 Hz. 

Following temporal feature extraction, CBAM is embedded by leveraging both channel and spatial attention mechanisms, since CBAM is lightweight and effective at strengthening the feature representation ability of the network. CBAM plays a vital role in handling the complex spatiotemporal dependencies of EEG data by adaptively enhancing the feature maps along the channel and spatial dimensions. Through its channel attention mechanism, CBAM adaptively emphasizes the most important feature maps by selectively highlighting relevant feature channels. Moreover, the spatial attention mechanism refines the spatial distribution of features within each feature map, capturing critical spatial patterns. A skip connection is included to ensure that important features from the temporal convolution are preserved and enhanced, further improving the feature extraction pipeline. 

The CBAM is composed of channel and spatial attention mechanisms with sequential arrangements. The intermediate feature map is defined as $F={\mathbb{R}}^{C\times H\times W}$; the attention process is summarized as follows:

(6) $$F^{\prime }=M_{c}(F)⊗F$$

(7) $$F^{\prime \prime }=M_{S}(F^{\prime })⊗F^{\prime }$$

where $M_{c}$ is the channel attention map, $M_{S}$ is the spatial attention map, $F^{\prime }$ denotes the refined feature map from channel attention, and $F^{\prime \prime }$ is the final refined output feature map. Channel attention operates via average-pooling and max-pooling simultaneously to obtain two spatial context descriptors $F_{\mathrm{avg}}^{C}$ and $F_{\max}^{C}$, and both are fed to a shared multilayer perceptron (MLP) network. The MLP consists of two fully connected layers, where the hidden layer reduces the channel dimension by a ratio of 16, followed by a ReLU activation, and the output layer restores the original channel dimension. The channel attention $M_{c}$ is calculated as follows:

(8) $$\begin{array}{c} M_{c}(F)=\sigma (MLP(AvgPool(F))+MLP(MaxPool(F)))\\ =\sigma (W_{1}(W_{0}(F_{\mathrm{avg}}^{C}))+W_{1}(W_{0}(F_{\max}^{C}))) \end{array}$$

where σ represents the sigmoid function and $W_{0}$ and $W_{1}$ are the weights of the MLP.

Based on the channel-refined feature map, the spatial attention is employed to obtain deeper refined features by focusing on the significance of ‘where in the interspatial areas of the feature map. The average-pooling and max-pooling operations along the channel dimension of the feature map generate feature descriptors $F_{\mathrm{avg}}^{S}$ and $F_{\max}^{S}$, which are subsequently concatenated and passed to a convolutional layer with a kernel size of 7 and padding of 3. Finally, the spatial attention $M_{s}(F)$ is computed as follows:

(9) $$\begin{array}{c} M_{s}(F)=\sigma (f^{7\times 7}([AvgPool(F);MaxPool(F)]))\\ =\sigma (f^{7\times 7}([F_{\mathrm{avg}}^{S};F_{\max}^{S}])) \end{array}$$

Overall, CBAM helps to enhance the representation of critical EEG features by adaptively emphasizing informative features. Through channel and spatial attention mechanisms, CBAM adaptively refines the input feature maps by multiplying the attention and dynamically weighing them based on both channel and spatial importance. 

Next, spatial convolution is executed with 40 filters of size (*C*, 1), where *C* represents electrode channels. Spatial features are extracted from the refined feature maps. Then, batch normalization is utilized to improve the training efficiency and model stability, and average-pooling along the temporal dimension and a dropout strategy are performed to prevent overfitting; the hyperparameters are detailed in Table 1. Finally, a safe log calculation is adopted to increase numerical stability. The final features obtained are the output of this module.

### 3.3. Classifier Module

A classifying convolutional layer with four filters of size (1, 11) is used as the classifier, and the output *M*-dimensional vector is computed via the SoftMax function expressed as follows:

(10) $$P_{i}=\frac{e^{z_{i}}}{\sum_{j=1}^{M}e^{z_{j}}}$$

where *M* is the number of MI task categories and $z_{i}$ is the score of the *i*-th category. Subsequently, cross-entropy loss is applied as the loss function for model training, which is formulated as follows:

(11) $$L=-\frac{1}{N_{b}}\sum_{i=1}^{N_{b}}\sum_{c=1}^{M}y\log(\overset{\wedge }{y})$$

where $N_{b}$ denotes the sample number for each batch, and y and $\overset{\wedge }{y}$ denote the real and predicted labels, respectively.

Totally, the preprocessed EEG data are fed to the model, and sequentially passed through the temporal refining module to obtain the refined EEG signals. The refined signals go through the attention-enhanced convolutional module to acquire refined EEG features, followed by the classifier module, which outputs the classification results. 

## 4. Experiments and Results

To assess the efficacy of the proposed model, an EEG collection experiment and MI decoding were conducted to classify multiple lower-limb MI tasks. The experiments were noninvasive and approved by the Ethics Committee of Shenzhen University (protocol number: PN-202300149). Ten subjects signed informed consent forms. In addition, the proposed model is also implemented using a public MI-EEG based BCI Competition (BCIC) IV 2a [54] dataset to assess model generalizability.

### 4.1. Experimental Protocol and Data Acquisition

#### 4.1.1. Participants

Ten physically and mentally healthy subjects (six males and four females, aged 18 to 34 years) have participated in this experiment. One day prior to the experiment, the subjects were informed to maintain adequate sleep and refrain from drinking alcohol. All the subjects have signed informed consent forms.

#### 4.1.2. Experimental Protocol

**Figure 3:**
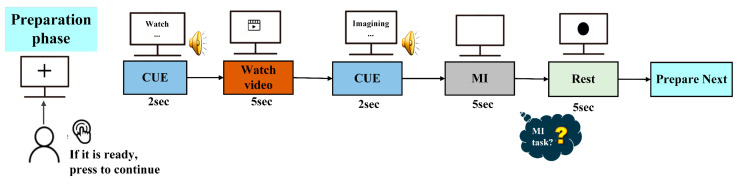
Experimental protocol (including the preparation phase, stimulus phase of watching ME video, MI phase, and rest phase).

In the MI-EEG data collection experiment, the visual stimulus is used to guide subjects through the implementation process of the specified MI task, as dynamic instructive guidance for MI is better for improving MI classification accuracy than picture guidance [55]. The experimental protocol is designed with reference to [56,57], as shown in Figure 3. The protocol consists of four phases: preparation, stimulus, MI, and rest. During the preparation phase, attention training is conducted. When the subject is ready, a button is pressed to enter formal MI experimentation. In the stimulus phase, a cue indicating the start of a visual stimulus appears. Then, a 5 s video is played. In the MI phase, a prompt displaying an MI task corresponding to the last visual stimulus reminds the subjects of their tasks; then, the subject imagines the task until it concludes within 5 s. The MI task comprises the following four LLM patterns (LLMPs): sit-to-stand (S-ST), stand-to-sit (ST-S), walking, and standing. Each task is randomly arranged in a run. During the rest stage, the subject is instructed to relax without thinking about any motor-related or extraneous matters. After each run, a random question answering mechanism is used to check the mental state of the subjects. The experimental protocol is implemented using E-Prime v 2.0.10.

Next, a suitable visual stimulus is presented to guide the subject in performing the specified MI task. Inspired by biological motion [58], we imitate the movement process with stick figures to produce motion videos for MI guidance. This approach helps to eliminate interference information such as color and environment details, thus enabling the subject to focus on capturing the primary information of the ongoing MI task and generating purer EEG data in the next stage. To execute the four representative LLMPs and abstract acceptable stick figure-like movement videos as MI guidance, the following steps are taken: (1) A clean and pure room is selected, and light-emitting diodes (LEDs) are placed at different positions on both the lower and upper limbs for a participant wearing black clothing. (2) The participant is asked to perform four LLM tasks in cycles as follows: ST-S, ST-S, walking, and standing. The entire motor execution (ME) process is captured using a camera. (3) The raw MI videos are then processed into binary black-and-white videos with each LED light transformed into a white spot using OpenCV-Python 4.8.1.78.

**Figure 4:**
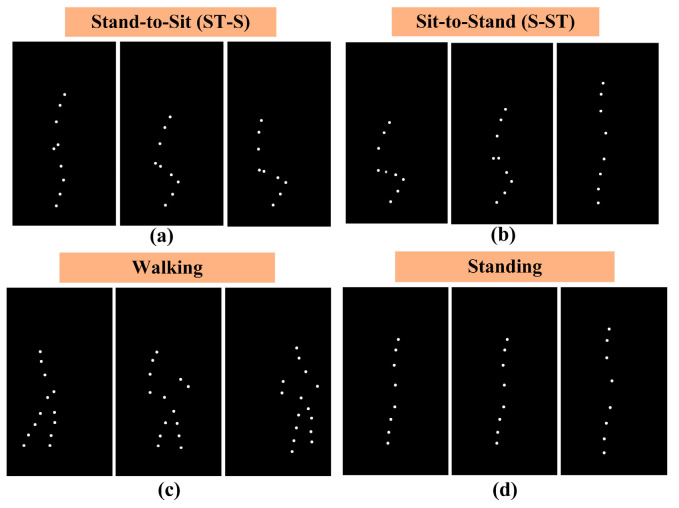
Stimulus video presentation: (**a**) ME process from standing to sitting down; (**b**) ME process from sitting to standing; (**c**) ME process of walking; (**d**) ME process of standing.

Two types of black-and-white comparison videos of the four ME tasks are created to make the rendering easier to understand, namely Comparison I and Comparison II. In Comparison I, the number of light points on the stick figure is consistent with each joint of the upper and lower limbs. Comparison II presents more light points on the stick figure when additional LED lights are attached between the ankle and hip joints during filming. A questionnaire investigating subjective feedback after watching the two types of ME videos is subsequently performed with 10 volunteers who have no prior knowledge of these raw ME tasks. Over 90% of the volunteers preferred Comparison II because of its vividness with increased visible movement from additional light points. The movement videos for S-ST and ST-S in Comparison I are difficult to identify as motion patterns of sitting and standing up. On the basis of the investigation results, we choose videos from Comparison II as the visual stimulus in this MI collection experiment. Finally, the MI guidance videos are obtained as displayed in Figure 4, reducing the impact of environmental interference factors to enhance the stimulus effect. 

#### 4.1.3. Data Acquisition Procedure

**Figure 5:**
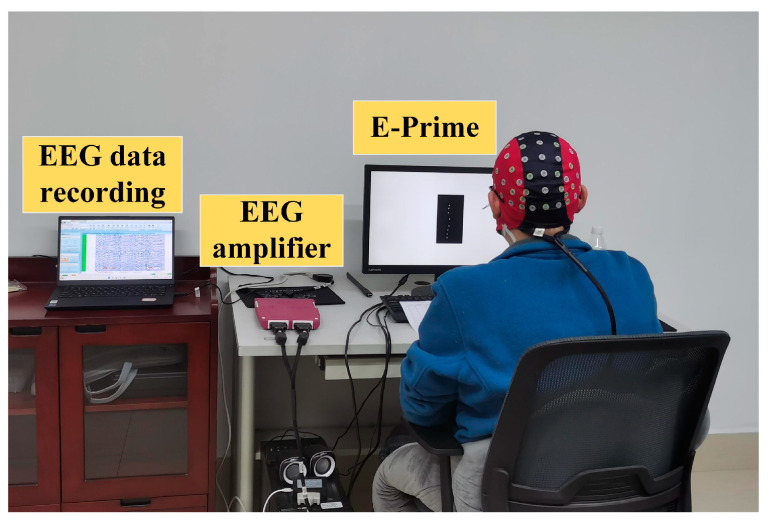
The experimental platform.

A quiet and independent laboratory is selected for the experiment. The experimental setup is depicted in Figure 5, with the hardware consisting of two computers for recording EEG data and running E-Prime and EEG collection equipment. The EEG collection equipment used is an ANT eego™ mylab 88 instrument (ANT Neuro, Berlin, Germany) equipped with 64 EEG electrodes adhering to the international 10–20 system. These electrodes are Fp1, Fpz, Fp2, F7, F3, Fz, F4, F8, FC5, FC1, FC2, FC6, M1, T7, C3, Cz, C4, T8, M2, CP5, CP1, CP2, CP6, P7, P3, Pz, P4, P8, POz, O1, O2, EOG, AF7, AF3, AF4, AF8, F5, F1, F2, F6, FC3, FCz, FC4, C5, C1, C2, C6, CP3, CP4, P5, P1, P2, P6, PO5, PO3, PO4, PO6, FT7, FT8, TP7, TP8, PO7, PO8, and Oz. For the EEG cap, CPz is the online reference channel, and GND is the grounding channel near Fpz. The sampling rate is set to 1000 Hz. The experimental procedure is as follows:

- The EEG collection apparatus is set up as shown in Figure 5. Essential materials such as conductive gel, syringes, and alcohol are prepared, and the subjects are arranged to wash and dry their hair to ensure optimal conditions for electrode placement.
- Basic information (e.g., name, age, weight, height) of the subjects is recorded, and they are informed of the experimental procedures and tasks.
- Conductive gels are injected into each electrode channel until the electrode impedance is less than 20 kΩ to improve data quality. The subject is instructed to perform experimental tasks (four MI tasks) according to the protocol displayed on the monitor, with EEG data and marker data recorded synchronously by eegoTM software version 1.10.0 in real time.
- The subjects have sufficient rest time between sessions to adjust their mental state accordingly. Each subject underwent 20 recording sessions within one month; each session included 60 trials.

#### 4.1.4. Data Preprocessing

The MI-EEG trials are with C electrode channels and T time points. The EEG data are recorded with 64 electrode channels. The raw data are easily contaminated by various artifacts, such as movement artifact, environmental noise, extracranial physiological noise, and electronic interference. Thus, a preprocessing regimen is essential to ensure the integrity and reliability of the data. The following preprocessing steps are implemented via the Python MNE library: (1) EEG segments ranging from 0.5 s to 5.5 s are extracted based on predefined event annotations; each segment contains 5000 time points, and the total data are labeled for categorization. (2) The electrooculography (EOG) channel is directly removed, and the remaining EEG data are re-referenced to the average of the bilateral mastoid electrodes (M1 and M2), resulting in a final dataset consisting of 61 EEG channels per trial. (3) A fifth-order Butterworth bandpass filter of 7–31 Hz is applied to eliminate extraneous noise and preserve task-relevant rhythms. (4) The data with a sampling rate of 1000 Hz (5000 time points per trial) are downsampled to 250 Hz (1250 time points per trial) to optimize computational efficiency while retaining task-relevant frequency components. Each trial is then divided into five samples (dimension of 61 × 250) for data augmentation using the sliding window technique [7], which helps to increase data samples. (5) The preprocessed EEG data are labeled to categorize and prepare for model training and analysis. (6) Minmax normalization, as shown in (12), is executed to reduce data fluctuations and nonstationarity.

(12) $$\tilde{x}=\frac{x-min(x)}{max(x)-min(x)}$$

where $\tilde{x}$ and x represent the normalized and input signals, respectively. 

### 4.2. Performance Metrics

The two most common metrics for classification accuracy (ACC) and the Kappa coefficient [1,59] are used to assess the classification performance. ACC is the ratio of the correctly predicted quantity to the total predicted quantity, offering a straightforward indicator of the model’s predictive performance. The Kappa coefficient is useful for addressing imbalanced data and random classification effects because it accounts for chance agreement, providing a more balanced measure of model performance. Kappa measures the ACC based on a confusion matrix (CM). The ACC and Kappa are defined as follows:

(13) $$Accuracy(ACC)=\frac{TP+TN}{TP+TN+FP+FN}$$

where *TP*, *FP*, *TN*, and *FN* represent the number of correctly predicted positive samples, incorrectly predicted positive samples, correctly predicted negative samples, and incorrectly predicted negative samples, respectively.

(14) $$Kappa=\frac{P_{0}+P_{e}}{1-P_{e}}$$

where *P_e_* denotes chance consistency, and *P_0_* is the accuracy.

Next, the CM provides detailed insight into the classification performance across different classes, and it reveals how well the model performs in predicting different classes. To statistically compare the proposed model with other comparison models, we employ the Wilcoxon signed rank test [60], a nonparametric test well-suited for small sample sizes and non-normally distributed data, to evaluate the statistical significance of performance differences across various methods. This ensures the robustness of the superiority of the proposed model. Specifically, a test is performed on the classification accuracies of MACNet and the baseline models for each subject. We defined a *p* value less than 0.05 as indicating a statistically significant difference.

### 4.3. Evaluating Generalizability

To evaluate the generalizability of the proposed MACNet, we implement it using the benchmark public MI-EEG dataset of the BCIC IV 2a. The dataset contains nine subjects with four MI tasks, left hand, right hand, feet, and tongue. Two sessions (288 trials/session) of EEG data were acquired with a sampling rate of 250 Hz for each subject, and the electrode number is 22 channels. The first session is the training set, and the second session is the test set.

### 4.4. Implementation Details

The proposed model is evaluated on the lower-limb MI dataset using a subject-dependent approach. The dataset includes four MI classes, with each class containing an equal number of trials per session to maintain a balanced class distribution. The MI-EEG data for each subject are merged and randomly divided into 10 equal groups for tenfold cross-validation. In each fold, 90% of the data are used for training and 10% for testing, and the model performance is averaged over all folds [1,61]. 

The proposed MACNet is compared with six advanced MI-EEG classification models: FBCSP [62], deep ConvNet [35], EEGNet [37], FBCNet [36], ATCNet [6], and Conformer [17]. We reproduced all the comparison models with hyperparameters in accordance with their original published articles. The data preprocessing, training, and evaluation steps follow the same procedure as that of the proposed model for fairness. Then, an ablation study on the lower-limb MI dataset is performed to evaluate the performance of each module in MACNet.

The MI-decoding models are built via Python 3.9.18 with the PyTorch 2.1.0 library. The models are trained and evaluated using an NVIDIA GeForce RTX 4090 GPU. For all the experiments, the Adam optimizer is used with a learning rate of 0.0001, and a learning rate scheduler based on the cosine annealing strategy is used to adjust the learning rate. The batch size is 64, the cross-entropy loss is set as the loss function, and an exponential moving average model is constructed to smooth the model parameters during training for robustness enhancement.

### 4.5. Overall Performance

A subject-dependent experiment is conducted on the lower-limb MI dataset to validate the effectiveness and evaluate the performance of the proposed MACNet model. The performance of MACNet is compared with that of six advanced MI-EEG decoding models. For the comparison models, the FBCSP [62] uses handcrafted spatial EEG features and previously won the BCI Competition IV. Many SOTA DL-based models, including deep ConvNet [35], EEGNet [37], FBCNet [36], ATCNet [6], and Conformer [17], were inspired by the FBCSP. Deep ConvNet incorporates five convolutional layers and a Softmax layer, and it was specifically designed to classify oscillatory signals. EEGNet performs a two-step convolution from the temporal domain to the spatial domain to learn frequency and spatial features; then, separable convolution is employed to reduce parameter counts and decouple feature map relationships. EEGNet is robust in learning various features across different BCI paradigms. FBCNet extracts spectro-spatial features from multi-frequency-band signals, making it less susceptible to small training sets. ATCNet and Conformer are the existing advanced transformer models in EEG decoding. ATCNet recently showed excellent feature extraction ability and obtained the highest classification performance on BCIC IV 2a. Conformer effectively decoded oscillatory EEG signals in subject-specific experiments. These comparison models have achieved impressive performance on public EEG datasets and are considered the backbone in the BCI field.

Table 2 summarizes the classification performance of each subject and the total average results, comparing the proposed MACNet with other models on the lower-limb MI dataset with respect to accuracy and Kappa. MACNet achieves the highest classification accuracy and Kappa among all the models, with an ACA across all the subjects of 98.54% and an average Kappa of 0.98. This model yields significant improvements over FBSCP (*p* < 0.005), EEGNet (*p* < 0.005), FBCNet (*p* < 0.005), deep ConvNet (*p* < 0.005), ATCNet (*p* < 0.005), and Conformer (*p* < 0.005) by 43.79%, 41.87%, 41.64%, 2.99%, 8.85%, and 11.17%, respectively. Deep ConvNet yields better results than the other comparison models do. Regarding the other SOTA models, ATCNet, which incorporates TCN and attention mechanisms, achieves 89.69% ACA; Conformer, which combines convolutional layers with transformer blocks, achieves 87.38% ACA. Both achieve relatively high ACAs on ten subjects, as they both extract local EEG features and learn global correlations from the temporal domain, resulting in better classification performance than that of pure CNN-based models (EEGNet and FBCNet) and the traditional FBCSP model. The results of ATCNet and Conformer reveal that transformer-based models are effective in MI-EEG decoding. However, transformers need larger datasets to realize their full potential. Alternatively, the proposed MACNet extracts refined local and global EEG features from both the temporal and spatial domains by integrating multidimensional attention mechanisms with ConvNet, leading to superior results and significant upgrades in terms of accuracy and Kappa. The results obtained by ATCNet align with those reported in [63], where a transformer-based EEG decoding model achieved an ACA of 97.33 ± 1.86% in LLMI recognition. Additionally, statistical analysis via the Wilcoxon signed-rank test confirms that the differences between the proposed MACNet and all the comparison models are statistically significant (*p* < 0.05), further demonstrating the robustness and effectiveness of the proposed model. 

**Figure 6:**
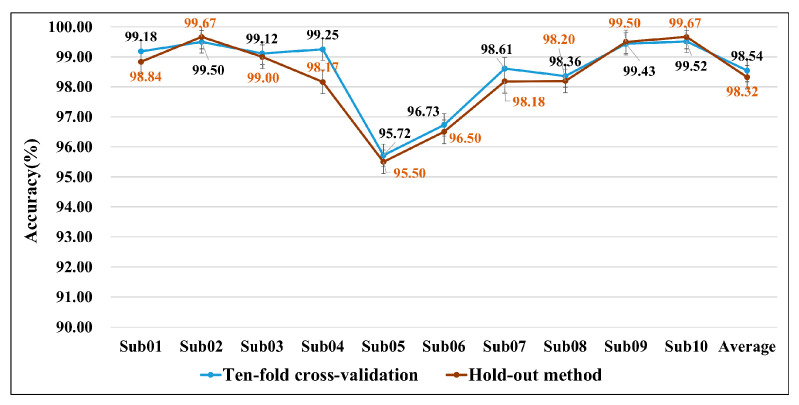
Classification accuracy of the proposed model on the basis of two experiments.

An extensive experiment is carried out to validate the robustness of the proposed MACNet via the hold-out method. The data of one random fold are used as the test set, while the data from the other nine folds serve as the training set. Figure 6 illustrates the classification accuracy for each subject based on the hold-out method and tenfold cross-validation method. The classification accuracy obtained using the hold-out method is closer to the results obtained under the tenfold cross-validation method, and the variation trends of classification accuracy based on the two experiments are similar. The results demonstrate that MACNet is robust for lower-limb MI classification.

### 4.6. Ablation Study

**Figure 7:**
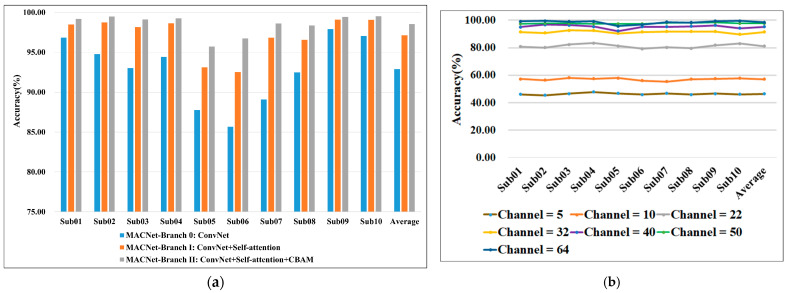
(**a**) Ablation study of MACNet; (**b**) Classification results of MACNet under channel reduction.

Ablation studies are performed on the lower-limb MI dataset to investigate the impact of different modules and the robustness of MACNet under varying channel configurations. MACNet is enhanced over ConvNet by the incorporation of temporal self-attention for reconstructing EEG signals and CBAM for refining EEG features across channel and spatial dimensions. The model branches are defined as follows: MACNet-Branch 0 serves as the baseline ConvNet, MACNet-Branch I consists of ConvNet and a temporal self-attention layer, and MACNet-Branch II is composed of ConvNet, a temporal self-attention layer, and CBAM. The ablation study results are presented in Figure 7. From Figure 7a, the classification accuracy gradually improves from MACNet-Branch 0 to MACNet-Branch II. The temporal self-attention mechanism helps in focusing on the most salient temporal characteristics and ignores less important details, and CBAM captures complex spatiotemporal dependencies in EEG data, demonstrating that both temporal self-attention and CBAM are successful in improving MI-EEG classification. Additionally, channel reduction analysis of Figure 7b demonstrates MACNet’s robustness with fewer channels; as the number of channels increases, the performance also improves.

These findings confirm the effectiveness of MACNet’s multidimensional attention mechanism in achieving robust feature extraction and classification across varying input and configuration conditions.

### 4.7. Visualization

To show the interpretability of the MACNet model, visualization results of the CM, t-distributed stochastic neighbor embedding (t-SNE)-based method mapping of high-dimensional features, and EEG topography are illustrated. 

**Figure 8:**
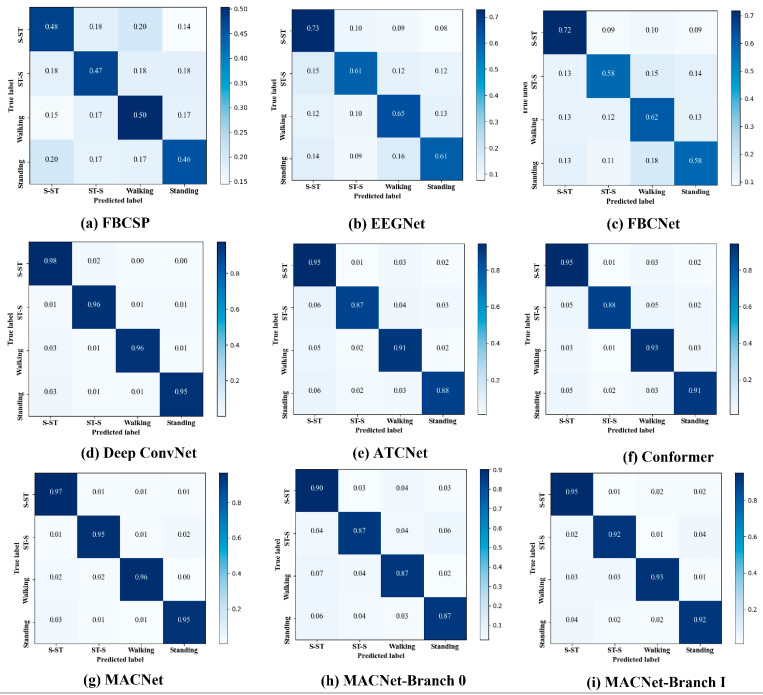
Average confusion matrices of the FBSCP, EEGNet, FBCNet, deep ConvNet, ATCNet, Conformer, proposed MACNet, MACNet-Branch 0, and MACNet-Branch I models.

The average CMs of MACNet and the reproduced models of subject 5 are shown in Figure 8. MACNet yields the best ACA in MI classification for all four MI classes, which is better than those of the other six models. Moreover, Figure 8g,h,i show the CM of MACNet and its branches 0 and I, respectively. The classification accuracy of each class is greater than that of the branch modules. The effectiveness of the proposed MACNet is further demonstrated.

**Figure 9:**
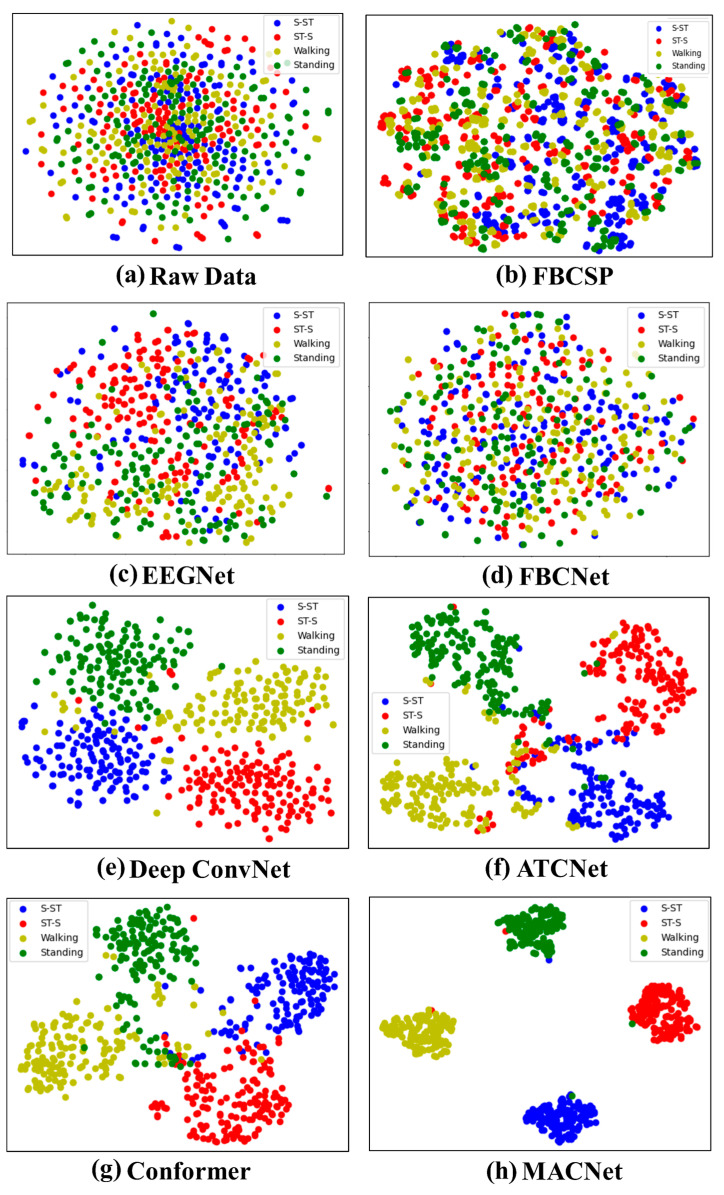
t-SNE visualization of learned EEG features using different methods; different colored dots represent different categories.

In addition, t-SNE is applied to visualize the feature distribution in a two-dimensional embedding space. Embedding helps to evaluate the discriminative power of high-dimensional features extracted by the proposed MACNet. As depicted in Figure 9, the distribution of the raw data is mixed. Compared with those of the FBCSP, EEGNet, FBCNet, deep ConvNet, ATCNet, and Conformer models, the features of different categories of MACNet are more separable. The effects of deep ConvNet, ATCNet, and Conformer are the second greatest. The aliasing between categories is evident in FBCSP, EEGNet, and FBCNet, where the features of different categories are relatively close. Figure 9h shows that the intercategory distance increases and the intracategory distance decreases, suggesting that the attention mechanisms in MACNet enable the extraction of more discriminative features. For example, separation in MI tasks such as walking and standing is particularly noticeable, where the brain activity patterns are more complex and difficult to distinguish. This finding indicates that MACNet can learn the most discriminative EEG features in lower-limb MI tasks.

**Figure 10:**
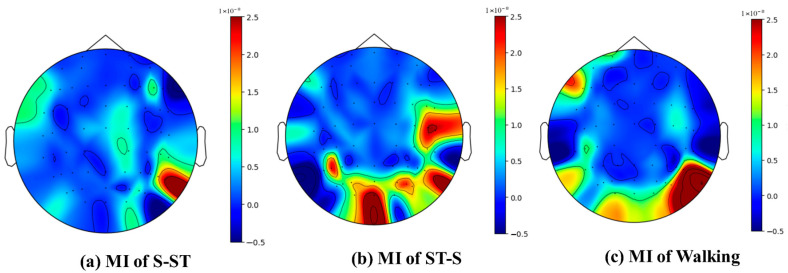
Brain average PSD topography maps for MI tasks. Blue indicates desynchronization while red indicates synchronization.

In previous studies [64,65], the imagination of standing was used as a baseline. Topographies of PSD differences (S-ST minus standing, ST-S minus standing, and walking minus standing) are displayed in Figure 10, depicting the PSD changes in brain areas through differences in characteristic activation values of each electrode channel across frequency bands ranging from 7 to 13 Hz (located in the mu band [8]). PSD is decreased in the mu band over the motor and sensory areas for the S-ST, ST-S, and walking MI tasks compared with the standing MI. These findings indicate that when the sensorimotor cortex of the brain is activated, event-related desynchronization (ERD) occurs in the mu rhythm. Which validates the neurophysiological relevance of the collected lower-limb MI-EEG data and aligns with established patterns in MI research.

### 4.8. Generalizability of the Proposed Model

For the performance on the widely used public dataset BCIC IV 2a, the classification accuracies of the proposed MACNet and comparison models are presented in Table 3. The accuracy achieved by MACNet is superior to that of FBCSP (*p* < 0.005), EEGNet (*p* < 0.05), FBCNet (*p* < 0.005), and deep ConvNet (*p* < 0.05), confirming its robust performance across different subjects. Although MACNet does not achieve the highest ACA, it is statistically comparable to the SOTA methods ATCNet and Conformer. The observed differences between these models’ performances are not statistically significant, as confirmed by the Wilcoxon signed-rank test (*p* > 0.05). This finding suggests that MACNet provides competitive performance on par with the best existing methods. Note that while ATCNet and Conformer require over 1000 training epochs to achieve optimum, MACNet achieves comparable accuracy with fewer than 500 epochs, demonstrating its efficiency in MI classification. This suggests MACNet is not data-dependent in MI-EEG decoding.

The results indicate that MACNet not only achieves statistically significant improvements in accuracy over most comparison models but also demonstrates comparable performance to SOTA methods, suggesting its robustness and generalizability in MI classification tasks.

## 5. Discussion

### 5.1. Effectiveness of MACNet

This paper presents MACNet for the exploration of multiple lower-limb MI classifications. The attention mechanism is generally incorporated from multiple dimensions such as channel, temporal, spatial, branch, or hybrid dimensions to improve network performance. MACNet integrates multidimensional attention mechanisms with ConvNet, including a temporal refining module and an attention-enhanced convolutional module, to obtain highly significant features from MI-EEG signals, thus enhancing the representative ability of the network. ConvNet comprises temporal and spatial convolution layers, where temporal convolution with a small kernel size captures high-frequency features, and spatial convolution operates along the electrode channels to extract spatial features, with the kernels acting as spatial filters [8]. The temporal refining module plays a crucial role in reducing noise from the raw EEG signals, enhancing the model’s ability to handle the low SNR associated with lower-limb MI tasks. The attention-enhanced convolutional module incorporating CBAM refines the feature maps along both the channel and spatial dimensions. This mechanism helps the model dynamically focus on task-relevant features while ignoring less significant features. The CBAM combines channel attention, which emphasizes “what” is important in the feature map, and spatial attention, which highlights “where the critical features are located. By adaptively recalibrating the attention weights, MACNet demonstrates better discrimination capability across different lower-limb MI tasks in terms of feature distribution, as shown in Figure 9. This superior feature separation contributes to the model’s improved classification accuracy, particularly in MI tasks with higher complexity. 

**Figure 11:**
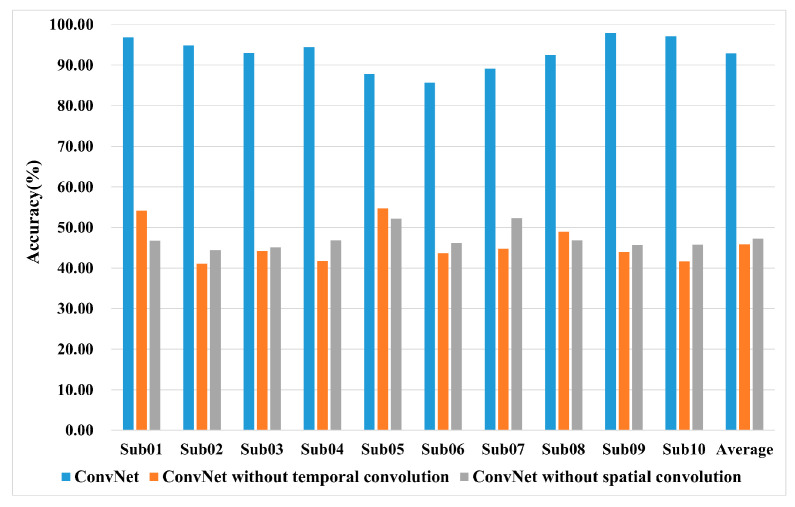
Classification accuracy of the baseline ConvNet model.

For the lower-limb MI task design, we choose four general LLMPs, namely, S-ST, ST-S, walking, and standing, for research on LLMI recognition. Most published studies on lower-limb MI decoding focus on classifying tasks, such as left-leg and right-leg movements, walking and relaxation, and walking and turning. Table 4 summarizes the comparison between the proposed model and those of previous works for LLMI recognition. The classification accuracies of different models are compared, and the proposed model offers advantages from a comprehensive perspective. Traditional ML techniques are commonly utilized to classify lower-limb MI tasks. This paper studies the multidimensional attention-based ConvNet model for decoding multiple lower-limb MI tasks and achieves remarkable results in subject-specific classification. We attribute this excellent result to two points: (1) the attention mechanism directs where to focus and improves the representation ability of interests, thus improving the classification performance; and (2) the baseline framework ConvNet performs well. Notably, we can see that deep ConvNet is also based on ConvNet and obtains outstanding classification accuracy, as listed in Table 2. When the ConvNet framework is separated into modules (ConvNet without temporal convolution, ConvNet without spatial convolution) to classify MI-EEG signals, the classification accuracy in Figure 11 shows that the overall ConvNet works better than when some modules are lacking, and removing the CNN module primarily affects the model’s ability to capture localized temporal and spatial patterns. In addition, the cross-validation approach may overrate accuracy among all EEG decoding models [66].

MACNet is validated via comparison experiments and ablation studies. The comparative results in Table 2 reveal that MACNet outperforms the SOTA models in terms of classification performance on the lower-limb MI dataset, such as EEGNet, FBCSP, FBCNet, deep ConvNet, ATCNet, and Conformer, across all subjects. Among these advanced comparison models, EEGNet is a general EEG decoding model that yields relatively low classification performance, possibly due to preprocessing issues. Deep ConvNet performs reasonably well, achieving high accuracy in some cases. However, its performance is inferior to that of MACNet; this can be explained by deep ConvNet’s lack of attention mechanisms, which limits its ability to selectively focus on the most informative channels and time periods. MACNet achieves superior classification performance compared with ATCNet and Conformer; this may be due to the action of multidimensional attention mechanisms in MACNet. The excellent classification performance can be attributed to MACNet’s advanced attention mechanisms, which adaptively focus on both spatial and temporal features, allowing it to handle the inherently noisy and complex EEG signals associated with lower-limb MI tasks. The confusion matrices (Figure 8) provide further insights into the classification performance of different models. Compared with the other models, MACNet results in significantly fewer misclassifications across all four lower-limb MI tasks (S-ST, ST-S, walking, and standing). For example, EEGNet and FBCSP exhibit greater confusion between tasks such as walking and standing, likely due to their inability to fully capture the subtle differences in brain activity associated with these similar tasks. In contrast, MACNet achieves extremely high classification in these tasks, which can be attributed to its attention-enhanced ability to distinguish task-relevant features, as well as the noise reduction provided by the temporal refining module.

The ablation study in Figure 7 clearly indicates that both temporal self-attention and CBAM contribute to the MACNet model. Specifically, MACNet-Branch II consistently outperforms MACNet-Branch I and MACNet-Branch 0 across all the subjects. This result highlights the roles of the temporal refining module and CBAM in capturing global patterns, complementing the local feature extraction capability of the ConvNet module. This enables MACNet to effectively handle the inherently noisy and complex spatiotemporal dependencies of EEG signals in lower-limb MI tasks. 

Figure 10 shows that S-ST, ST-S, and walking imagery tasks activate the sensorimotor cortex of the brain and induce ERD in mu rhythm, demonstrating neurophysiological patterns characteristic in MI. This corresponds with previous psychological findings [2,72,73,74]. This consistency validates the scientific credibility of the experimental data and reinforces its relevance for studying MI classification. Furthermore, the subtle PSD changes underscore the inherent difficulty in distinguishing lower-limb MI tasks based solely on traditional features such as PSD. Classifying lower-limb MI is an intuitive and natural way to detect HMI [75]. This differentiates MI-based systems from other EEG paradigms like SSVEPs or event-related potentials (ERPs), which are driven by external stimuli. For instance, the CSP approach was utilized to enhance spatial filtering for MI classification, focusing on optimal feature selection [75]. However, CSP methods primarily address spatial domains, whereas MACNet’s multidimensional attention mechanism dynamically captures both spatial and temporal dependencies. For other EEG paradigms, ERP classification was studied by using a multiscale convolutional network (MOCNN) [76], and ERP signals are event-related potentials that are time-locked to specific sensory or cognitive stimuli. This structured nature allowed the MOCNN to capture local and global temporal features effectively using multiscale convolutional layers. Unlike these paradigms, MI lacks external event triggers, requiring more sophisticated methods to capture subtle spatial and temporal dependencies in the EEG data. Thus, the proposed MACNet extends the concept of multiscale convolution by incorporating attention mechanisms and a multidimensional feature extraction method to address the more diffuse and complex nature of MI signals. MACNet is specifically designed to address lower-limb MI classification, which shows promise for application in user-friendly BCI systems, such as lower-limb exoskeletons (LLEs), wheelchairs, and prostheses, where the real-time, accurate detection of MI is essential for the practical human–machine interaction.

### 5.2. Model Complexity Analysis

In addition to classification accuracy, computational efficiency is crucial for real-time BCI systems. Thus, we evaluate the computational overhead and complexity of MACNet by comparing the number of parameters, floating point operations (FLOPs), and inference time with those of other SOTA models in the MI-EEG classification. The comparison results of different models with a data batch size of 64 are listed in Table 5, and the inference time is averaged over 100 runs based on Intel(R) Pentium(R) Gold G5420 and NVIDIA GeForce RTX 4090. As shown in Table 5, MACNet incorporates additional attention modules compared with shallow ConvNet, resulting in slightly higher parameter counts and a marginal increase in FLOPs. For the proposed MACNet, the inclusion of the temporal refining module and CBAM increases the computational cost because of the additional operations in the temporal, channel, and spatial dimensions. However, the corresponding increase in inference time is minimal, indicating that the attention mechanisms significantly enhance feature extraction without substantially compromising real-time performance. Compared with other SOTA models, MACNet maintains a competitive balance between model complexity, computational overhead, and real-time feasibility. Although some models, such as EEGNet and FBCNet, offer lower computational costs, they do so at the expense of classification accuracy. More complex models, like ATCNet and Conformer, offer high accuracy but also substantially increase higher computational costs. On the other hand, MACNet, by integrating multidimensional attention mechanisms, achieves higher accuracy with only a marginal increase in computational demand, making it highly suitable for real-time BCI applications. Overall, the trade-off between computational complexity and accuracy is carefully managed in MACNet, ensuring that it maintains high accuracy while being computationally feasible for practical real-time BCI applications [77].

### 5.3. Limitations and Future Directions

Although the proposed MACNet achieves superior performance for LLMI classification, several limitations remain that can be improved in the future. 

The relatively small size of the lower-limb MI-EEG dataset may affect the model’s generalizability. We employed a data augmentation technique, including a nonoverlapping sliding window approach, to increase the dataset size. This method segments continuous EEG signals into multiple smaller windows, significantly expanding the training data and reducing the risk of overfitting. However, we recognize that sliding window augmentation alone may not be sufficient to fully overcome the limitations posed by small datasets. Other data augmentation techniques, such as adversarial training and synthetic data generation, could be explored to further enhance model robustness. In our previous research, we experimented with generative adversarial networks (GANs) to generate synthetic EEG data [78], which yielded promising results in increasing data diversity and improving model performance on public EEG datasets. Nevertheless, the GAN-based approach is effective in augmenting data but introduces a significant computational overhead simultaneously. As a result, we choose the traditional sliding window technique for data augmentation in LLMI classification, considering the computational constraints. We also validated the proposed model on a public MI-EEG dataset and obtained competitive classification performance, further confirming its generalizability. 

EEG signals exhibit considerable individual differences and high variability from session to session [8]. Intersubject variability remains challenging in EEG-based MI classification. Differences in brain anatomy, cortical activation patterns, and signal quality across participants affect model generalizability. Although MACNet’s multidimensional attention mechanisms have demonstrated adaptability to subject-specific features, it is limited in learning useful information from other subjects, further improvements are required to address intersubject variability. Future work could explore additional strategies, such as subject-independent training methods and transfer learning that enable the rapid adaptation to new subjects, to promote the universality of the model and reduce the calibration time in real-time BCI systems. 

Although MACNet has been validated on benchmark datasets, cross-validation and experiments with larger and more diverse lower-limb MI-EEG datasets will also be considered to further validate the robustness and universality of this model. MACNet is an end-to-end framework that directly integrates feature extraction and classification into a unified pipeline. Although it currently relies on preprocessed EEG signals, future work will focus on integrating preprocessing steps, such as artifact removal and bandpass filtering, directly within the network to increase practicality and adaptability in real-world BCI applications. Additionally, exploring channel selection or reduction techniques is another avenue to prioritize the most informative EEG channels, thereby optimizing computational efficiency and facilitating deployment in resource-constrained BCI systems.

## 6. Conclusions

This paper proposed a multidimensional attention-based convolutional network, named MACNet, for lower-limb MI-EEG classification. MACNet integrates a temporal refining module and an attention-enhanced convolutional module. A specified lower-limb MI-EEG dataset comprising ten subjects is constructed, including MI tasks of the following four general LLMPs: S-ST, ST-S, walking, and standing. The effectiveness of MACNet was evaluated on the lower-limb MI dataset through comparative experiments and ablation studies. The results demonstrated that the proposed MACNet outperformed the state-of-the-art models in terms of classification accuracy and Kappa, and each module of MACNet enhanced the model’s representation ability and improved the classification performance. In addition, visualization analysis revealed the characteristics of feature learning and potential relationships between LLMI and brain activity. The generalizability of the model is further validated on a public benchmark EEG dataset. Overall, MACNet shows promising performance in advancing subject-specific lower-limb MI classification. This work provides a reference for the development of BCI systems for lower limb auxiliary or rehabilitation applications. 

## Figures and Tables

**Table 1:** The architecture of the proposed model.

Block	Layer		Kernel Size	Stride	Output Shape	Parameter Setting
Input	Input				[B, 1, C, 250]	B = 64, C = 61
Temporal refining module	Query and key projection				[B, 250, C]	head_num = 1, head_dim = 61
	Attention score				[B, 1, C, 250]	
	Concatenation				[B, 250, C]	
	Residual add				[B, 1, C, 250]	
Attention-enhanced convolutional module	Temporal convolution		(1, 25)	(1, 1)	[B, 40, C, 226]	
	CBAM-channel attention	AdaptiveAvgPool &AdaptiveMaxPool			[B, 40, 1, 1]	channel_input = 40, ratio = 16
		Multilayer perceptron	(1, 1)	(1, 1)	[B, 40, 1, 1]	
		Residual add			[B, 40, 1, 1]	
	CBAM-spatial attention	Average-pooling and max-pooling			[B, 40, 1, 11]	
		Concatenation			[B, 1, C, 226]	
		Convolution	(7, 7)	(1, 1)	[B, 4, 1, 1]	padding = 3
		Residual add			[B, 40, 61, 226]	
	Spatial convolution		(C, 1)	(1, 1)	[B, 40, 1, 226]	
	Batch normalization				[B, 40, 1, 226]	
	Average pooling		(1, 75)	(1, 15)	[B, 40, 1, 11]	
	Drop out				[B, 40, 1, 11]	0.3
Classifier	Convolution		(1, 11)	(1, 1)	[B, 4]	

B = batchsize, C = number of channels, head_num is number of heads, head_dim is dimension of each head, channel_input is the input feature channels in CBAM, ratio is the ratio when compressing the feature channels.

**Table 2:** Classification accuracy and Kappa of the different models on the lower-limb MI dataset.

Sub.	Proposed Model (MACNet)		FBCSP		EEGNet		FBCNet		Deep ConvNet		ATCNet		Conformer	
	ACC	Kappa	ACC	Kappa	ACC	Kappa	ACC	Kappa	ACC	Kappa	ACC	Kappa	ACC	Kappa
Sub01	**99.18**	**0.99**	59.73	0.32	57.20	0.43	64.88	0.53	96.29	0.95	88.04	0.84	93.58	0.91
Sub02	**99.50**	**0.99**	52.11	0.31	54.63	0.40	54.82	0.40	95.25	0.94	89.30	0.86	68.70	0.58
Sub03	**99.12**	**0.99**	52.47	0.32	53.95	0.39	52.58	0.37	93.27	0.91	87.05	0.83	93.53	0.91
Sub04	**99.25**	**0.99**	55.61	0.32	57.43	0.43	53.80	0.38	96.77	0.96	88.67	0.85	94.07	0.92
Sub05	95.72	0.94	58.20	0.31	65.00	0.53	62.47	0.50	**96.22**	**0.95**	90.18	0.87	91.58	0.89
Sub06	**96.73**	**0.96**	50.58	0.31	50.33	0.34	57.32	0.43	93.73	0.92	93.00	0.91	68.55	0.58
Sub07	**98.61**	**0.98**	55.99	0.32	57.87	0.44	56.82	0.42	97.01	0.96	91.54	0.89	84.46	0.79
Sub08	**98.36**	**0.98**	58.08	0.31	56.97	0.43	57.86	0.44	95.64	0.94	87.98	0.84	93.98	0.92
Sub09	**99.43**	**0.99**	52.01	0.32	55.85	0.41	53.77	0.38	95.65	0.94	91.93	0.89	97.98	0.97
Sub10	**99.52**	**0.99**	52.73	0.31	57.45	0.43	54.77	0.40	95.73	0.94	89.23	0.86	87.33	0.83
Mean	**98.54**	**0.98**	54.75	0.32	56.67	0.42	56.91	0.42	95.56	0.94	89.69	0.86	87.38	0.83
St.D.	1.23	0.02	3.02	0.00	3.53	0.05	3.78	0.05	1.15	0.02	1.84	0.02	10.03	0.13

The best results are bolded.

**Table 3:** Classification accuracy on BCIC IV 2a.

	Sub 01	Sub 02	Sub 03	Sub 04	Sub 05	Sub 06	Sub 07	Sub 08	Sub 09	Mean	St.D.
FBCSP *	65.05	54.86	62.85	57.52	54.05	52.43	56.48	61.11	57.87	58.02	4.21
EEGNet *	77.43	53.47	89.58	65.63	73.61	61.11	75.35	74.31	71.53	71.33	10.35
FBCNet *	72.26	50.60	87.38	57.38	68.33	50.18	76.96	80.83	78.93	69.21	13.59
Deep ConvNet *	76.04	50.00	82.99	72.22	73.96	56.25	79.86	75.00	76.74	71.45	10.97
ATCNet *	81.25	**61.81**	92.36	76.04	**79.17**	**71.53**	78.82	85.42	82.99	78.82	8.66
Conformer *	**89.24**	59.03	92.01	**81.60**	65.63	64.24	**94.10**	86.11	**85.42**	**79.71**	13.19
**Proposed model (MACNet)**	85.76	59.72	**93.75**	71.18	68.40	59.03	87.15	**85.07**	83.33	77.04	12.72

* Reproduced; the best result is highlighted in bold.

**Table 4:** Comparison between the proposed model and previous methods for LLMI recognition.

Ref.	Methods	Lower-Limb Motion Intention	ACCs
Kwak et al. [67]	Canonical correlation analysis method for feature extraction and k-nearest neighbors for classification	Classify LLMIs of walking, turning left or right, sitting, and standing, based on EEG signals.	91.3 ± 5.73%
Wu et al. [68]	CSP for feature extraction and SVM for classification	Classify LLMIs of standing, walking, and sitting, based on EEG signals.	92.06%
Wang et al. [69]	CSP for feature extraction and SVM for classification	Classify LLMIs of standing up, sitting down, and walking forward, based on EEG signals, and biomechanical signals of angle and ground reaction force.	94%~97%
Song et al. [70]	Hierarchical neural network	Classify LLMIs of standing, sitting, walking, ST-S, and going up and down stairs, based on EEG and surface electromyography(sEMG) signals.	65.07%
Shi et al. [71]	A dense coattention mechanism-based multimodal enhance fusion networks model	Classify LLMIs of standing, sitting, and walking, based on EEG and sEMG signals.	82.96%
	Proposed model	Classify LLMIs of S-ST, ST-S, walking, and standing, based on EEG signals.	98.54 ± 1.23%

**Table 5:** Parameter number, number of FLOPs, and inference time comparison.

Model	Params (K)	FLOPs (MMac)	Inference Time (ms)
MACNet	111.92	41.30	1.96
Shallow ConvNet	100.44	35.88	0.48
EEGNet	2.53	8.15	0.80
FBCNet	2.69	1.02	0.82
Deep ConvNet	144.8	1.69	2.16
ATCNet	128.524	117.11	2903.70
Conformer	340.22	37.92	12.96

## Data Availability

Data are unavailable due to privacy and ethical restrictions.
